# Relationships among the Characteristic Tensile Strain, Curing Age, and Strength of Reactive Powder Concrete

**DOI:** 10.3390/ma14102660

**Published:** 2021-05-19

**Authors:** Min Guo, Ri Gao

**Affiliations:** School of Civil Engineering, Beijing Jiaotong University, Beijing 100044, China; rigao@bjtu.edu.cn

**Keywords:** reactive powder concrete (RPC), initial tensile strain, ultimate tensile strain, age, elastic modulus, characteristic age, compressive strength, tensile strength

## Abstract

The characteristic tensile strain of reactive powder concrete is a critical indicator of its resistance to cracking. In order to study its crack resistance performance, in this study, we investigated changes over time in the characteristic tensile strain patterns of reactive powder concrete. An axial tensile test was performed to obtain the stress–strain curves of reactive powder concrete after curing ages from 3 to 56 days, and then we identified changes over time in the initial and ultimate tensile strain patterns. An analysis was conducted to determine the correlation between the initial tensile strain and the ratio of tensile strength to elastic modulus. The correlations between the ultimate tensile strain and its curing age as well as that of the ultimate tensile strain with its tensile strength and its compressive strength were established, and an approach was proposed for calculating the characteristic age of reactive powder concrete.

## 1. Introduction

Reactive powder concrete (RPC) is a fiber-reinforced cement-based composite material that exhibits ultra-high strength, high toughness, high volume stability, high durability, and low permeability [1,2]. By enhancing the micro and macro characteristics of an RPC mixture, uniformity, the largest particle packing density, and the smallest defect size in the material can be achieved [3,4]. Characterized by ultra-high strength, toughness, and durability, RPC has the potential to significantly improve the performance of cement-based materials. Compared with ordinary concrete and even high-performance concrete (HPC), both the compressive strength and tensile strength of RPC along with its toughness are significantly enhanced [5]. Conventional concrete exhibits weak tensile strength, which is commonly ignored when applied to structures, and therefore steel bars and other materials are used to remedy this defect. There is a considerable number of steel fibers uniformly distributed in an RPC matrix, enabling RPC to maintain high tensile strength after being pulled and cracked [6,7]. Therefore, the notably improved tensile properties of RPC are the key indicators that distinguish RPC from other HPC. In addition to emphasizing the applications of the ultra-high compressive strength of RPC, the specifications of RBC all highlight the neglected tensile properties of conventional concrete materials; the axial tensile performance of RPC is considered to be the critical index of its structural design [8].

The traditional methods for testing the tensile properties of concrete are an axial tensile test, flexural test, and splitting tensile test. Due to the obvious brittleness of ordinary concrete, a simple and easy flexural test is often used to test its tensile performance. For RPC, tensile stress and strain can be determined using an axial tensile test because of an obvious increase in toughness. In addition, an axial tensile test can reflect the tensile properties of materials more intuitively, and therefore, current studies on the tensile properties of RPC have mainly been conducted using an axial tensile test. Although there is currently no unified standard for an RPC axial tensile test, numerous tests have been conducted with many results [9]. Tensile strength and strain, elastic modulus, and dissipated energy of RPC have been shown to be positively proportional to the steel fiber content, curing age, and loading rate [10,11]. A tensile constitutive relationship of RPC is also in the exploratory stage. Currently, widely accepted constitutive models are the piecewise curve model [12], the piecewise linear model [13], and the three-section linear model [14].

Regarding tensile properties, studies have routinely focused on crack resistance. The characteristic tensile strain of RPC is a priority of crack resistance research on RPC, and it is also treated as a major index that reflects the crack resistance performance of RPC. The characteristic tensile strain involves both initial crack strain and ultimate strain, which represent the tensile strain when RPC develops cracks and fails, respectively. By conducting a series of tests, Kaplan concluded that tensile strain could play a more significant role in the initial cracking of concrete than tensile stress [15]. Tasdemir [16] argued that the ultimate axial tensile strain of concrete exhibited a typical linear relationship with the ratio of axial tensile strength to elastic modulus, regardless of the type, size, or gradation of the aggregate and how the test was performed, the length o the strain gauge, curing age, as well as the load application rate also had little effect on this result, suggesting that the application of strain as the judgment criterion would be more effective for predicting RPC cracking.

Additionally, the current superior mechanical properties of RPC are largely determined by heat treatment [17,18,19]. Although there have been extensive studies on the application of RPC in various areas of the field of engineering (e.g., houses, bridges, and railways), defects in steam curing conditions and accompanying high cost have severely restricted widespread promotion of RPC [20]. Accordingly, in this study, we propose curing RPC using a standard curing method [21] for ordinary concrete and we focus on the effects of curing age on the tensile properties of RPC. Previous studies have shown that the compressive strength as well as the stress at initial cracking increase as curing age increases and that a significant portion of this increase takes place within the first 28 days after casting [22,23]. Ultimate tensile strength and elastic modulus of strain-hardening cement-based composites (SHCC) increase with increasing curing age [10,24,25]. 

In this study, we designed a direct tensile test for RPC, and obtained the full tensile stress–strain curves of RPC under different curing ages. At the same time, the test results intuitively reflected the trends of characteristic tensile strain of RPC at different curing ages, and we summarized the relationships among curing age, compressive strength, tensile strength, and characteristic tensile strain; therefore, laying a foundation for studies on RPC crack resistance. 

## 2. Experimental Program

### 2.1. Production of Test Materials and Test Samples

**Figure 1:**
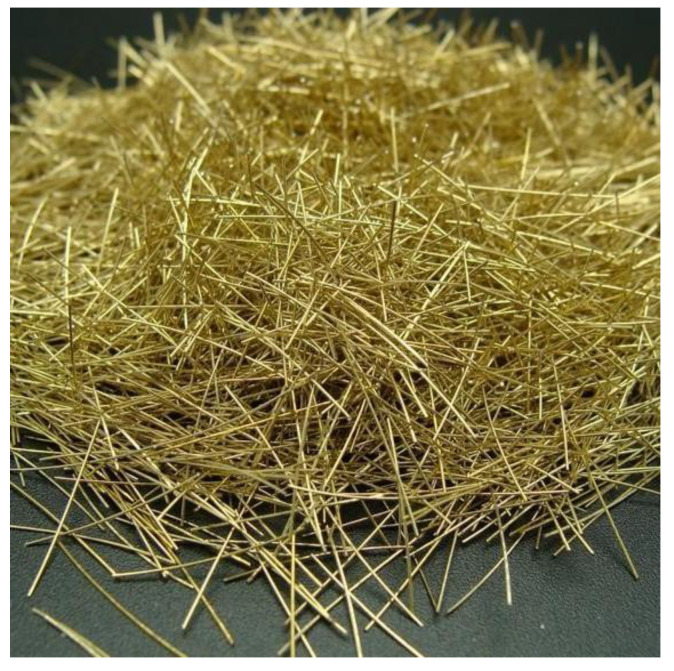
Steel fiber for the RPC axial tensile test.

Ordinary Portland cement (PO42.5, SanHeJiDong Cement Co., Ltd., Langfang, China) was used in this study. The initial setting time reached 198 min, the final setting time was 264 min, the loss on ignition was 2.5%, the density was 3.06 g/cm^3^, the specific surface area was 390 m^2^/kg, the 28-day flexural strength was 8.6 MPa, and the 28-day compressive strength reached 51.4 MPa. Silica fume was high- and medium-quality micro-silica fume, and the color was dark gray. The SiO_2_ content was 95.12%, the density reached 2.2 g/cm^3^, the ignition loss was 2.4%, and the specific surface area was 19,000 m^2^/kg. The water content was 1.2% and the chloride ion content was 0.018%. The 28-day activity index was 124% and the water demand was 104%. The particle size of the quartz sand was in the range of 0.16~1.25 mm; on the basis of particle size, the quartz sand could be classified into three types, i.e., coarse, medium, and fine with particle sizes of 0.16~0.315, 0.315~0.63, and 0.63~1.25 mm, respectively. The water reducer was a polycarboxylic high-performance water-reducing agent (model CBMA-101B, China Building Materials Academy, Beijing, China). The steel fiber consisted of special thin round and short thin steel fibers, with copper-plated surfaces, a diameter of 0.22 mm, a length of 12~15 mm, and tensile strength of 2800 MPa (Figure 1). The water was tap water from a laboratory at Beijing Jiaotong University.

To satisfy the working performance, based on the reactive powder concrete mix ratio used by domestic scholars, five basic mix ratios were considered [8,26,27,28,29]. By altering the types of raw materials, the mixing amount, and the water/binder ratio, the mix ratio that satisfied the requirements was determined, as listed in Table 1.

The reactive powder concrete was prepared by mixing the dry materials, and then the wet materials as follows: First, the raw materials of the reactive powder concrete were weighed according to the mixing ratio. Then, the weighed quartz sand and steel fiber were poured into the concrete mixer and mixed for 2 min to evenly disperse the steel fiber in the quartz sand. Subsequently, cementitious materials (e.g., cement and silica fume) were added and stirred for 1 min to achieve full contact among the cementitious materials, quartz sand, and steel fibers and uniformly distribute them on a macroscopic scale. Finally, the water and high-efficient water-reducing agent were added to the mixer and stirred for 5–8 min. The workability of the mixed concrete was checked. The slump was measured according to the workability test method of ordinary concrete. The test results are shown in Table 2.

Next, the mixed reactive powder concrete was put into the sample molds at one time, and then vibrated on a vibrating table. The finished reactive powder concrete sample molds were placed in a standard curing room for 24 h, at ambient temperature, and then the molds were removed (standard curing refers to the curing conditions defined by the “Standard Test Methods for Concrete Physical and Mechanical Properties” GB/T50081-2019) [21]. The samples were placed in the standard curing room to reach the corresponding curing ages before being removed for testing. All of the preparation and curing methods of RPC were based on the Chinese national standard “Reactive Powder Concrete” (GB/T 31387-2015) [30].

**Figure 2:**
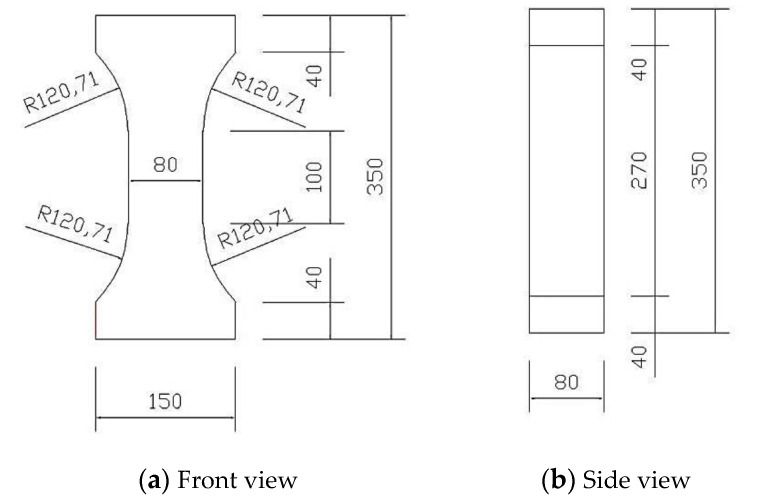
Dimensions of the RPC dumbbell-shaped test samples (mm).

In this study, the reactive powder concrete samples were classified into five groups according to the different curing ages. There were three samples tested in each group, i.e., a total of 15 samples. The curing method and curing age were set as standard curing for 3, 7, 14, 28, and 56 days. The samples for the axial tension test were dumbbell shaped. Given the large stress concentration of the dumbbell-shaped samples at the variable cross-section, to improve the stress state of the samples and to increase the probability that they break in the uniform stress section in the middle, the samples were designed to slowly transition from the middle tension zone to the end, and the transition section was arc shaped (Figure 2).

### 2.2. Test Method 

**Figure 3:**
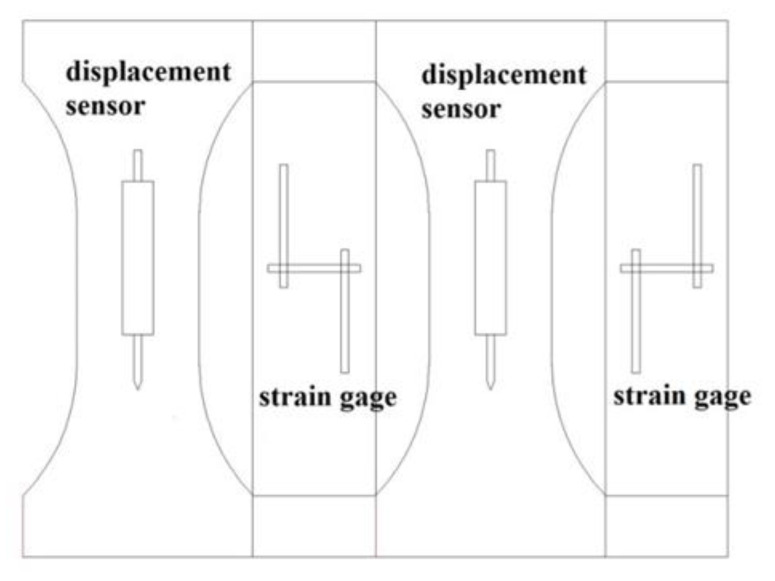
The location of the strain gauges attached on a sample.

**Figure 4:**
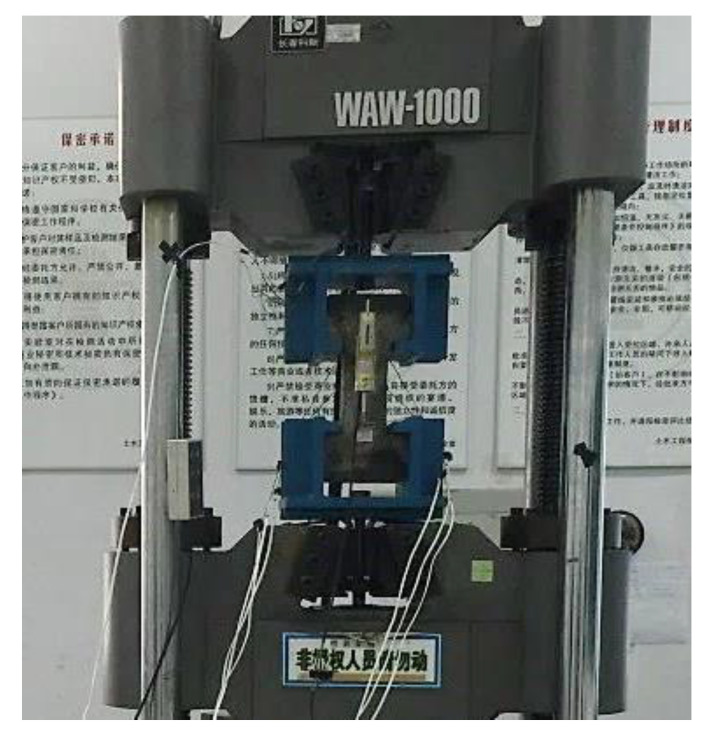
Diagram of loading device for the RPC axial tensile test.

The dumbbell-shaped samples were shaped by the pouring process, and the uniaxial tensile test was performed after reaching the curing age of the test design. Prior to the tensile test, two paper-based resistance strain gauges were pasted on two sides of the sample, respectively, and one displacement sensor was arranged on the other two sides of the sample, respectively, parallel to the axial direction. (Figure 3). The test was performed on a WA-1000 universal testing machine (Figure 4), supplemented by a static strain test system for dynamic collection of strain data.

## 3. Analysis of Experimental Results

### 3.1. Full Axial Tensile Stress–Strain Curves

**Figure 5:**
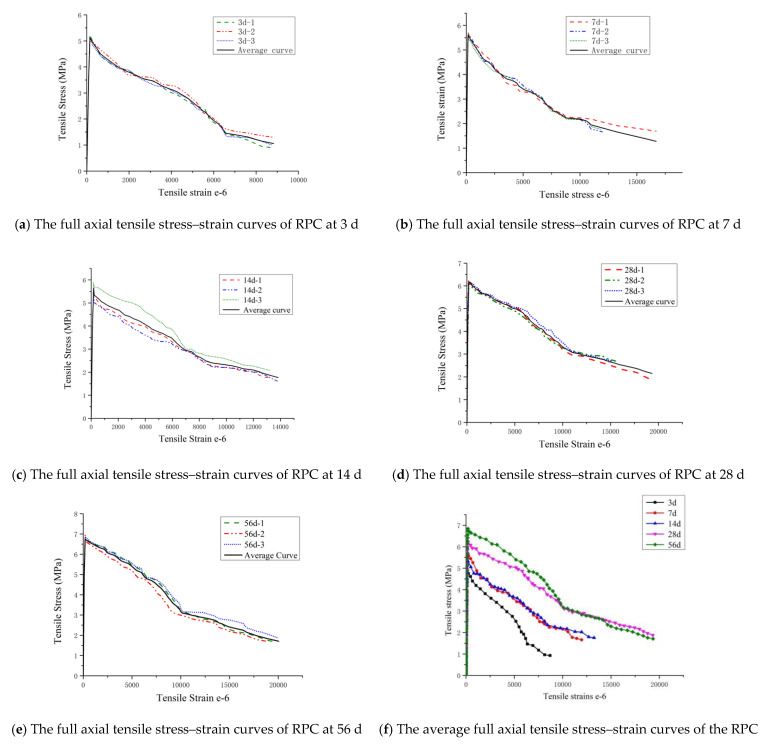
The full axial tensile stress–strain curves of the RPC at various curing ages.

Figure 5 shows the full axial tensile stress–strain curves of RPC at various curing ages, and it can be observed that the error of the uniaxial tensile test is in an acceptable range. From Figure 5f, it can be observed that the trend of the average stress–strain curves of RPC at all curing ages is basically the same, which means all curing ages undergo the elastic, strain hardening, and stress softening stages. Obvious differences were identified in the peak points of the curves at different curing ages, i.e., the tensile strength was obviously different. 

**Figure 6:**
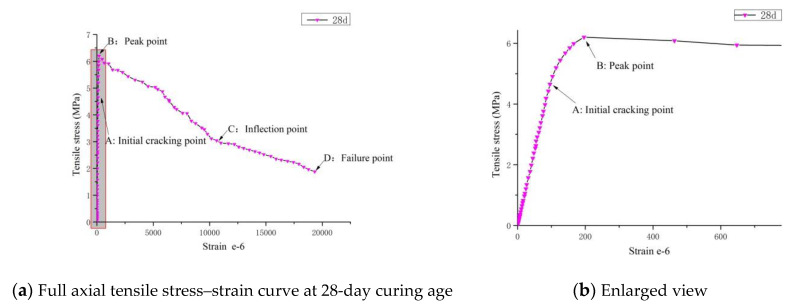
Full axial tensile stress–strain curve of RPC at 28-day curing age.

For example, consider the RPC at the 28-day curing age, as shown in Figure 6, point A represents the initial crack point in a general circumstance, at which the stress is about 75% of the ultimate stress and the strain is referred to as the initial crack tensile strain. According to Blakey [31,32], the initial tensile strain signals the development and expansion of micro cracks inside the concrete. Before point A is reached, the curve is approximately linear. At this time, the material is in the elastic stage. In this stage, the components begin to be stressed, and the internal micro cracks are at a relatively stable and independent development phase; the stress and strain show a linear increasing trend. Although steel fiber is incorporated, under the sidewall effect of the aggregate on the steel fiber, the steel fiber is distributed parallel to the sidewall of the aggregate, which does not restrain the development of cracks. Beyond point A, the curve increasingly deviates from a straight line, which suggests the occurrence of strain hardening. When the curve peaks at point B, the specimen reaches its limit in terms of load-bearing capacity. The stress corresponding to point B is the tensile strength of RPC, while the strain corresponding to point B is the ultimate tensile strain of RPC. Then, it enters the stress softening stage. According to Nicholas et al. [33], reaching the initial crack strain is a significant sign that the concrete risks imminent failure and the ultimate tensile strain indicates the maximum strain withstood by the concrete.

**Figure 7:**
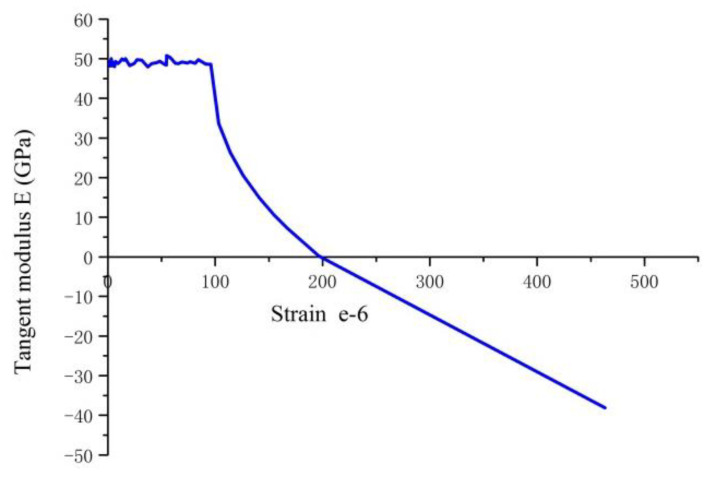
Axial tension strains, tangent elastic modulus curve of RPC.

In this study, the tangent elastic modulus method was applied to obtain the initial crack tensile strain and ultimate tensile strain of RPC. Figure 7 shows the relationship curve of tensile strain and tangent elastic modulus of RPC at 28-day curing age. It can be seen from the figure that the initial section of tangent elastic modulus is almost a straight line with some negligible fluctuations, and that the average value of this section represents the axial tensile elastic modulus of RPC. The strain corresponding to the intersection where the straight line begins to enter the declining curve is the initial tensile crack strain of RPC, while the strain corresponding to the intersection of the curve and the horizontal axis is the ultimate tensile strain of RPC.

The main results of the RPC axial tensile test are listed in Table 3. According to the data in the table, with an increase in curing age, the compressive strength and tensile strength of RPC increase significantly. The difference is that the compressive strength increases obviously with increasing curing age, while the tensile strength at 3 days reaches 83% of that at 28 days, and slowly increases at a later stage. The elastic modulus also increases with increasing curing age, about 81% of the elastic modulus at 28 d develops after 3 d, and slowly increases at a later stage. As compared with compressive strength, it is obvious that both tensile strength and the elastic modulus develop significantly at an early age. The initial tensile strain and the ultimate tensile strain both increase with curing age. Next, we discuss the relationship between the initial and ultimate tensile strains. 

### 3.2. Relationship between Initial Tensile Strain and Age

**Figure 8:**
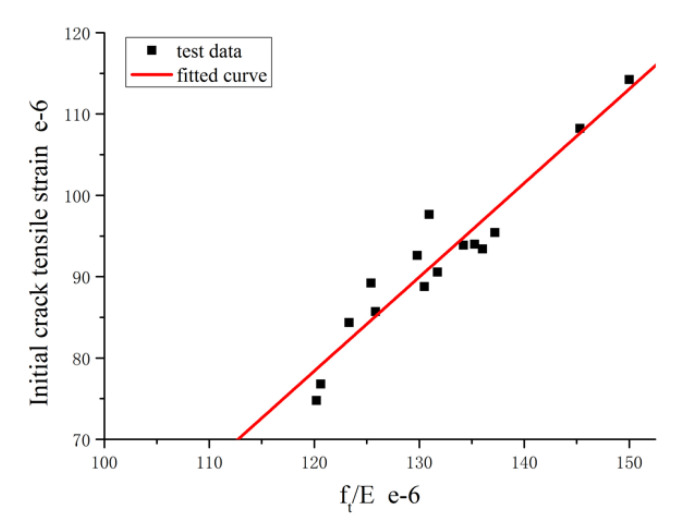
Relationship between initial crack tensile strain and $f_{t}/E$.

The material of the RPC specimens is in an elastic state after axial tensile stress is applied and before the initial tensile strain is reached. At this stage, the elastic theory is used to analyze the patterns of initial tensile strain of RPC that developed over time. Figure 8 shows the relationship between the initial crack strain of RPC at different curing ages and the ratio of tensile strength to tangent elastic modulus as established and indicated by [34]. It can be observed from the figure that the initial crack strain of RPC at different curing ages, *ε*_t,c_, shows a typical linear relationship with the ratio of tensile strength to tangent elastic modulus, *f_t_*/*E*. For the mix ratio used in this study, the relationship between the two is expressed as follows:

(1) $$\varepsilon_{t,c}\left(t\right)=a\cdot \frac{f_{t}\left(t\right)}{E\left(t\right)}+b$$

According to the test data, *a* = 1.16, *b* = $-60.28\times 10^{-6}$, and R^2^ = 0.904. According to the analysis of the obtained experimental data, it can be concluded that $\frac{f_{t}\left(t\right)}{f_{t}\left(28\right)}=0.167\;\ln t+0.434$, $E\left(t\right)=\frac{t}{0.156+0.201t}$, where *t* represents the ag.

### 3.3. Relationship between Ultimate Tensile Strain and Age

**Figure 9:**
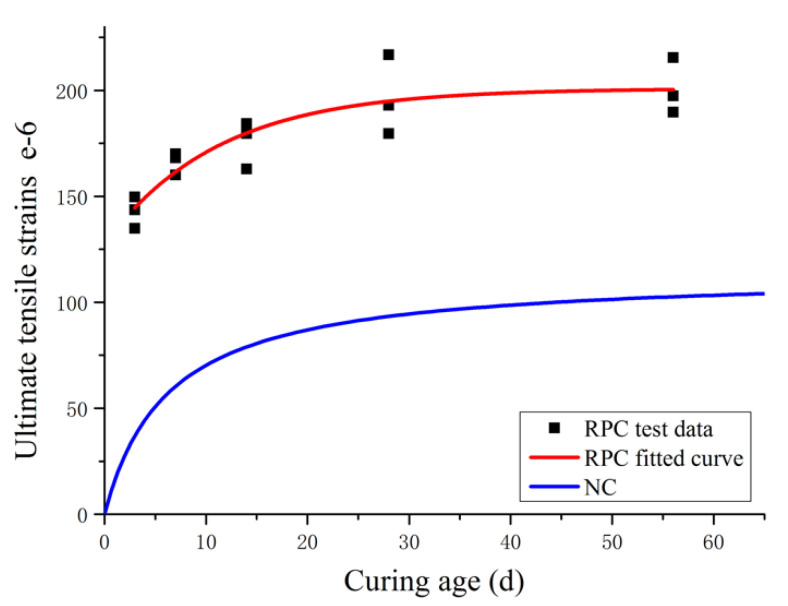
Relationship between ultimate tensile strain and curing age.

According to the results from previously conducted studies, it is known that the ultimate tensile strain of ordinary concrete increases as the curing age of concrete increases, similar to the relationship between ultimate tensile strain and curing age of RPC. According to the test data, the relationship curve between the ultimate tensile strain and curing age was established, as shown in Figure 9, and the relationship between the two can be expressed as follows, at R^2^ = 0.788:

(2) $$\varepsilon_{t,p}\left(t\right)=\varepsilon_{max}-ae^{-\frac{t}{b}}$$

where $\varepsilon_{max}$ indicates the measured maximum value of the ultimate tensile strain. 

According to the test data,$\;\varepsilon_{max}=215.46$. By fitting the experimental data, it can be obtained that *a* = 73.62, *b* = 11.17, and R^2^ = 0.788. Meanwhile, the relationship curve between the ultimate tensile strain and curing age of ordinary concrete (NC) was plotted according to the research results of M. F. Kaplan [15], as shown in Figure 9. By contrast, the ultimate tensile strains of both ordinary concrete and RPC increase with an increase in curing age, at a pace that is faster in the initial stage but moderate in the later stage. From an early curing age, the overall ultimate tensile strain of RPC is significantly greater as compared with ordinary concrete, and it increases rapidly. The ultimate tensile strain of RPC at a curing age of 3 days is nearly four times that of ordinary concrete. Then, the growth rate of the ultimate tensile strain for ordinary concrete increases progressively, especially before the curing age reaches 14 days, with a sharp increase observed. When the curing age reaches 28 days, the ultimate tensile strain of RPC is two times that of ordinary concrete. As a critical indicator of the crack resistance performance of RPC, the ultimate tensile strain of RPC determines the capability of RPC to restrict crack width. Thus, it can be concluded that RPC far outperforms ordinary concrete in terms of crack resistance and the earlier the curing age, the greater the advantage.

As shown in Figure 9, the ultimate tensile strain of RPC does not increase indefinitely with curing age. After a certain curing age is reached, the ultimate tensile strain basically converges to a fixed value. Despite a slight increase or fluctuation, the overall change is insignificant, as follows:

(3) $$\varepsilon_{t,p}\left(t\right)=\varepsilon_{\infty }\;t\geq t_{c}$$

where *t_c_* represents the age when the ultimate tensile strain basically reaches a fixed value, which is known as the characteristic age indicated by *d*; *ε*_∞_ refers to the ultimate tensile strain reached by RPC at the characteristic age, which is considered to be the ultimate tensile strain of RPC and represents the maximum value of crack resistance of RPC.

According to the test data, it is suggested that the relationship between RPC ultimate tensile strain and curing age can be expressed using the following formula:

(4) $$\varepsilon_{t,p}\left(t\right)=\varepsilon_{\infty }-ae^{-\frac{t}{b}}\;$$

where *a* and *b* are a constant. 

It can be seen from Equation (2) that *a* = 73.62 and *b* = 11.17. Then, the derivative of Formula (4) is calculated to obtain the rate that the ultimate tensile strain changes with age as follows:

(5) $$\frac{d\varepsilon_{t,p}}{dt}=\frac{a}{b}e^{-t/b}$$

From Formula (5), it is determined that the ultimate tensile strain of RPC declines on a continued basis with the growth rate of the curing age. When the growth rate is sufficiently low, it is considered that RPC has reached the characteristic age. In this study, an assumption is made that, when RPC reaches the characteristic age, the growth rate of its ultimate tensile strain is less than one micro strain per day ($1\times 10^{-6}/d$). On the basis of this, the expression of the characteristic age of the ultimate tensile strain can be expressed as follows:

(6) $$t_{c}=-b\ln\left(b/a\right)$$

The RPC characteristic age of the mixture ratio in this experiment was calculated to be 21 d.

### 3.4. Relationship between Ultimate Tensile Strain and Strength

#### 3.4.1. Relationship between Ultimate Tensile Strain and Tensile Strength

The ultimate tensile strain of ordinary concrete increases with an increase in tensile strength. According to the test data, RPC shows the same trend. In this study, it is suggested that the relationship between the ultimate tensile strain and tensile strength of RPC is expressed using the following formula:

(7) $$\varepsilon_{t,p}=\left(a\ln f_{t}+b\right)\times 10^{-6}$$

**Figure 10:**
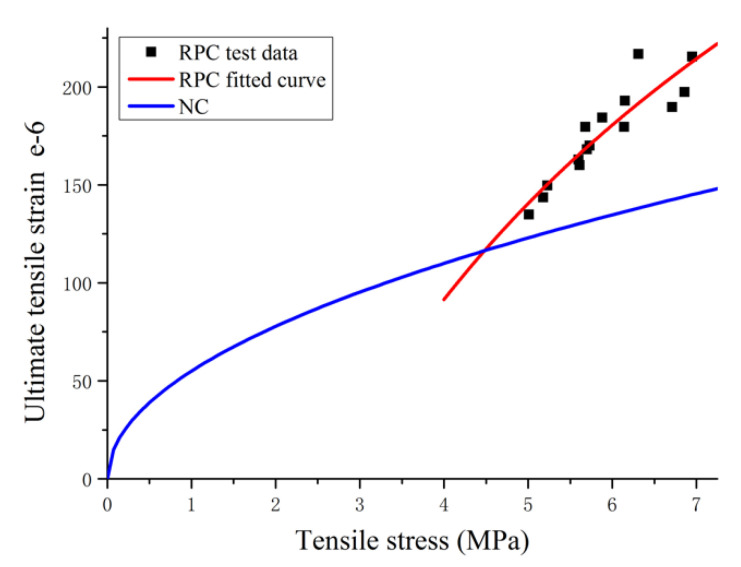
Relationship between tensile strength and ultimate tensile strain of RPC.

According to the test data, a = 219.48, b = −212.77, and R^2^ = 0.815. As shown in Figure 10, as compared with ordinary concrete [35], the relationship between the tensile strength and ultimate tensile strain of RPC is similar to that of ordinary concrete, with a proportional increase observed. As the tensile strength increases, however, the ultimate tensile strain of RPC increases at a faster pace and shows a linear relationship. When the tensile strength is identical, the ultimate tensile strain is significantly higher than ordinary concrete, indicating that the crack resistance of RPC is clearly superior to ordinary concrete.

#### 3.4.2. Relationship between Ultimate Tensile Strain and Compressive Strength

**Figure 11:**
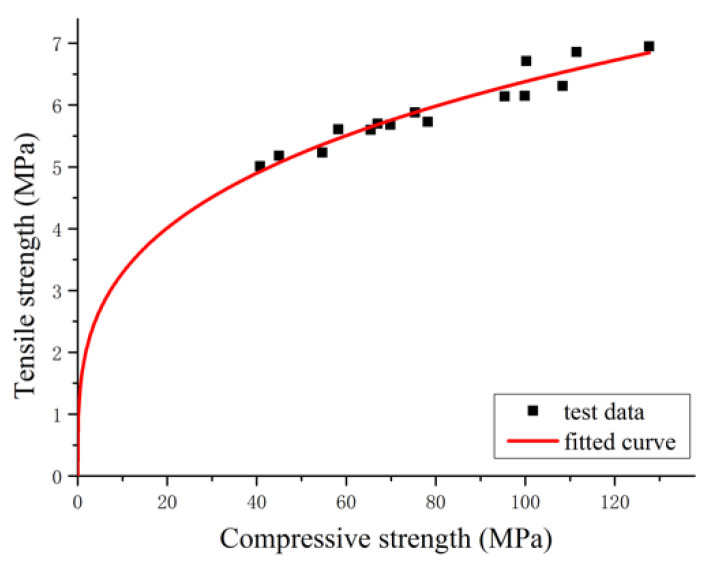
Relationship curve between tensile strength and compressive strength of RPC.

In order to establish the correlation between ultimate tensile strain and compressive strength, first, it is necessary to determine the relationship between compressive strength and tensile strength. According to the test data, the relationship curve between compressive strength and tensile strength of RPC at different curing ages is shown in Figure 11, with the formula of the two fitted as follows:

(8) $$f_{t}=af_{c}^{b}$$

where *f_t_* indicates the tensile strength of RPC, *f_c_* denotes the compressive strength of RPC, and *a* and *b* are a constant. According to the test data, *a* = 1.69, and *b* = 0.29, and R^2^ = 0.908.

With Formula (8) substituted into Formula (7), the relationship between the compressive strength and ultimate tensile strain of RPC can be obtained as follows:

(9) $$\varepsilon_{t,p}=\left(328+63.65\ln f_{c}\right)\times 10^{-6}$$

The ultimate tensile strain of RPC is considered to be a crucial index to use for the calculation of crack resistance exhibited by materials and is also considered to be a significant parameter for the design of mass concrete in terms of temperature control. When there is no test data available, it is essential to estimate the ultimate tensile strain. In this case, the above-mentioned relational expression can be used to estimate the known tensile strength or compressive strength, which is of practical significance to applications in the engineering field.

## 4. Conclusions

In this study, the direct tensile behavior of RPC for a series of five different curing ages were experimentally investigated using a direct tensile test, and some of the conclusions are as follows:

- The trend of changes in the axial tension stress–strain curves of RPC at all curing ages is similar, all the curves involve three stages, i.e., the elastic, strain hardening, and stress softening stages. There is an obvious difference in the tensile strength of RPC with curing age.
- There is a typical linear relationship between the axial tensile crack strain at different curing ages and the ratio of tensile strength to elastic modulus at the corresponding curing ages. According to the test data, the relationships between tensile strength and curing age and between elastic modulus and curing age are obtained, therefore, the relationship between tensile strain and curing age of initial crack could be established. The initial crack tensile strain of RPC is an important sign of material cracking; it directly reflects the crack resistance of the material. Therefore, in this study, we have shown the influence of curing age on the crack resistance of RPC.
- The ultimate tensile strain of RPC increases with an increase in the curing age and, at a late stage, becomes flattened. The ultimate tensile strain can reflect the crack width control ability of RPC. Compared with ordinary concrete, the crack width control ability of RPC has obvious advantages, and the earlier the curing age, the more obvious the advantage.
- A method for calculating the characteristic age of RPC is presented in this study. In addition, the relationships between RPC ultimate tensile strain and compressive strength and between ultimate tensile strain and tensile strength are proposed for estimating ultimate tensile strain exhibited by known RPC strength for applications in the field of engineering.

## Figures and Tables

**Table 1:** RPC test mix ratio.

	Cement	Silica Fume	Quartz Sand			Steel Fiber	Water Reducing Agent	Water
			Coarse	Medium	Fine			
**Dosage kg/m^3^**	920	170	460	340	280	120 ($V_{f}=2\%$) ^1^	22	174
**Mix ratio**	1	0.18	0.5	0.37	0.3		0.024	0.19

^1^ Where $V_{f}$ denotes the volume content of steel fiber.

**Table 2:** Slump test results for RPC.

Test Batch	Slump (mm)	Expand Slump (mm)	Conclusion
1	270	450	The workability is good, the mixer is slightly wet
2	260	408	The workability is good
3	270	410	The workability is good
4	280	490	The workability is good

**Table 3:** Main test results of the RPC axial tension test.

Age	Average Compressive Strength $f_{t}(\mathrm{MPa})/\mathrm{SE}$	Average Tensile Strength $f_{t}(\mathrm{MPa})/\mathrm{SE}$	Average Initial Tensile Strain $\varepsilon_{t,p}$ $(\times 10^{-6})/\mathrm{SE}$	Average Ultimate Tensile Strain $\varepsilon_{t,p}$ $(\times 10^{-6})/\mathrm{SE}$	Elastic Modulus $E_{t,0}\;(\mathrm{MPa})$
3 d	46.8/2.45	5.14/0.139	84.67/4.31	142.76/6.492	3.97 × 10^4^
7 d	67.8/2.44	5.68/0.143	92.00/5.65	166.10/8.672	4.55 × 10^4^
14 d	70.2/3.22	5.72/0.277	86.21/3.35	175.62/7.171	4.53 × 10^4^
28 d	101.2/5.08	6.2/0.076	95.77/2.92	196.44/6.648	4.88 × 10^4^
56 d	113.1/5.96	6.84/0.124	101.18/6.82	200.85/4.065	5.0 × 10^4^
